# Bottom-of-sulcus dysplasia: the role of ^18^F-FDG PET in identifying a focal surgically remedial epileptic lesion

**DOI:** 10.1186/s41824-020-00092-w

**Published:** 2020-12-15

**Authors:** S. U. Berlangieri, R. Mito, M. Semmelroch, M. Pedersen, G. Jackson

**Affiliations:** 1 Department of Molecular Imaging and Therapy, Austin Health, Melbourne, VIC Australia; 2 Florey Institute of Neuroscience and Mental Health, Melbourne, VIC Australia; 3 Department of Psychology and Neuroscience, Auckland University of Technology, Auckland, New Zealand; 4 Department of Neurology, Austin Health, Melbourne, VIC Australia; 5 Florey Department of Neuroscience and Mental Health, The University of Melbourne, Melbourne, VIC Australia

**Keywords:** Bottom-of-sulcus dysplasia, ^18^F-FDG PET, MRI, Focal cortical dysplasia

## Abstract

**Purpose:**

Bottom-of-sulcus dysplasia (BOSD) is a type of focal cortical dysplasia and an important cause of intractable epilepsy. While the MRI features of BOSD have been well documented, the contribution of PET to the identification of these small lesions has not been widely explored. The aim of this study was to investigate the role of F-18 fluorodeoxyglucose (^18^F-FDG) PET in the identification of BOSD.

**Methods:**

Twenty patients with BOSD underwent both ^18^F-FDG PET and structural MRI scans as part of preoperative planning for surgery. Visual PET analysis was performed, and patients were classified as *positive* if they exhibited a focal or regional hypometabolic abnormality, or *negative* in the absence of a hypometabolic abnormality. MRI data were reviewed to determine if any structural abnormality characteristic of BOSD were observed before and after co-registration with PET findings.

**Results:**

PET detected hypometabolic abnormalities consistent with the seizure focus location in 95% (19/20) of cases. Focal abnormalities were detected on ^18^F-FDG PET in 12/20 (60%) patients, while regional hypometabolism was evident in 7/20 (35%). BOSD lesions were missed in 20% (4/20) of cases upon initial review of MRI scans. Co-registration of ^18^F-FDG PET with MRI enabled detection of the BOSD in all four cases where the lesion was initially missed.

**Conclusion:**

Our findings show that ^18^F-FDG PET provides additional clinical value in the localisation and detection of BOSD lesions, when used in conjunction with MRI.

## Introduction

Bottom-of-sulcus dysplasia (BOSD) is a type of focal cortical dysplasia (FCD) in which the dysplastic abnormality is located at the depth of a sulcus. These small dysplastic lesions are clinically important, as not only are they focally epileptogenic, but their surgical removal results in excellent rates of seizure freedom (Harvey et al. 2015). While BOSD shares pathological features with type II FCD, it is commonly classed as a distinct subtype given that its size and localisation make it difficult to detect upon routine imaging. The imaging features of BOSD are therefore important to understand, as correct identification can result in an excellent post-surgical outcome.

The MRI hallmarks of BOSD have been well documented and include cortical thickening at the sulcal bottom, blurring of the grey-white matter boundary, abnormal signal intensity, and commonly including a transmantle sign (Harvey et al. 2015; Hofman et al. 2011). BOSDs also exhibit hypometabolism on fluorodeoxyglucose (^18^F-FDG) PET scans, suggesting that they contain no normal function (Harvey et al. 2015). While the role of PET in detecting BOSDs is suggested to be important, the contribution of PET to the identification of these small lesions has not been explored.

The aim of this study was thus to investigate the role of ^18^F-FDG PET along with MRI in the identification of BOSD in patients with focal intractable epilepsy.

## Methods

### Patients

Patients with drug-resistant epilepsy were identified from the Comprehensive Epilepsy Program (CEP) at Austin Health (Melbourne, Australia) between 2005 and 2016, searching initially for patients in whom BOSD was suggested from preoperative imaging. All patients underwent clinical workup, including clinical history, neuropsychology, electroencephalography (EEG)/Video EEG, MRI, SPECT, and PET leading to an offer of surgery in those patients considered suitable surgical candidates. For this study, only patients who proceeded to surgery were included.

BOSD was diagnosed on MRI as previously described (Harvey et al. 2015), based on the presence of cortical thickening and grey-white matter blurring that was maximal at the depth of a sulcus, with or without a transmantle sign and cortical and subcortical signal change. Only patients with a single BOSD were included, and only patients with FCD type II were included in this study.

Of the 35 initially selected patients, seven were excluded as they exhibited diffuse abnormality not confined to a single sulcus, four were excluded as they exhibited complex pathology or histological features other than FCD type II, three were excluded as they did not have PET data available at our site, and one was excluded as they did not proceed to surgery.

### Imaging data

All patients were imaged preoperatively at either 3 T on a Siemens Skyra or Trio scanner (*n* = 13) or at 1.5 T on a Siemens Avanto scanner (*n* = 7). The MRI protocol has been described previously (Hofman et al. 2011). The imaging protocol included acquisition of a volumetric T1-weighted sequence, axial and coronal T2-weighted sequences, volumetric fluid-attenuated inversion recover (FLAIR) sequence, and susceptibility-weighted and diffusion-weighted sequences.

^18^F-FDG PET was acquired on either a Philips Allegro PET (*n* = 13) or Philips TF64 PET/CT (*n* = 5) scanners (Philips Healthcare, Cleveland, OH). The Philips Allegro protocol included a 4- to 6-h fast followed by 250 MBq ^18^F-FDG administered intravenously in a dimly lit room and 30-min uptake period. The PET Brain study was acquired into a 256 × 256 matrix at 3 frames of 10 min each. Attenuation correction was performed using a Ge-68 germanium source. The Philips TF64 protocol was identical except for a 200 MBq ^18^F-FDG dose, single frame 15-min acquisition and CT attenuation correction. Two PET studies were acquired at external sites and details of their acquisition protocol not made available.

Preoperative MRI scans were reviewed for all patients by an epileptologist (G.D.J.) to identify any BOSDs and categorised as *positive* or *negative* based on the presence or absence of a focal lesion at the bottom of a sulcus, initially blinded to PET data.

Visual PET analysis was performed by a trained examiner (S.U.B) to identify any metabolic abnormalities, using a colour scale to detect variation in ^18^F-FDG uptake. Metabolic abnormalities were classified as *focal* (with demarcated margins compared to the adjacent cortex, and involving not more than two gyri), or as *regional* (exhibiting a gradual decline in activity compared to the adjacent cortex, and involving one or more lobar regions).

MRI and PET data were automatically co-registered using MedView v12 (MedImage Inc., Ann Arbor, MI). MRI data were then reviewed in light of PET, clinical and video-EEG findings. The visual PET findings were considered concordant with MRI data if the area of hypometabolic activity overlapped with the identified structural BOSD lesion.

### Histology

Cortical samples were taken from a surgical specimen, and histopathology slides were reviewed by a neuropathologist (R.M.K) to determine the FCD subtype.

## Results

### Patients

Twenty patients (11 males, 9 females) were included in the study, with a mean age at surgery of 32 (± 10.7) years (range 18–53 years). The dysplastic lesion was located in the right hemisphere in 12 patients and left hemisphere in 8 patients. With regards to lesion lobe, lesions were located in the frontal lobe in 11/20 (55%), parietal lobe in 6/20 (30%), insula in 1/20 (5%), frontotemporal in 1/20 (5%), and temporoparietal in 1/20 (5%) patients. Clinical data for each patient is provided in Table 1.

**Table 1:** Patient clinical data

Case	Sex	Lesion location	Pathology	Initial MRI	PET findings	Age	Surgical outcome
1	M	R frontal	FCD IIA	Pos	Regional	18	Infrequent seizures
2	M	R parietal	FCD IIB	Pos	Focal	18	Seizure free
3	M	L frontal	FCD IIB	Pos	Focal	38	Seizure free
4	F	R frontal	FCD IIB	Pos	Focal	23	Seizure free
5	M	R frontal	FCD IIB	Pos	Regional	39	Recurrent seizures
6	M	L parietal	FCD IIA	Neg	Regional	20	Seizure free
7	M	R frontal	FCD IIB	Pos	Focal	32	Seizure free
8	F	R parietal	FCD IIB	Pos	Focal	37	Seizure free
9	M	L frontal	FCD IIB	Neg	Focal	36	Seizure free
10	F	R frontal	FCD IIB	Pos	Focal	23	Recurrent seizures
11	F	L frontal	FCD IIB	Pos	Regional	23	Seizure free
12	F	R temporo-parietal	FCD IIB	Pos	Negative	37	Seizure free
13	M	L frontal	FCD IIB	Pos	Focal	53	Seizure free
14	M	R parietal	FCD IIB	Pos	Regional	25	Seizure free
15	F	R fronto-temporal	FCD IIB	Pos	Focal	42	Seizure free
16	F	R parietal	FCD IIB	Pos	Focal	28	Seizure free
17	F	R insula	FCD IIB	Pos	Regional	32	Seizure free
18	F	L parietal	Neg	Neg	Focal	18	Seizure free
19	M	L frontal	FCD IIB	Neg	Regional	49	Seizure free
20	M	L frontal	FCD IIB	Pos	Focal	45	Seizure free

### Imaging findings

MRI was considered positive in 16/20 (80%) of patients, and negative in four (20%) upon initial review.

**Fig. 1:**
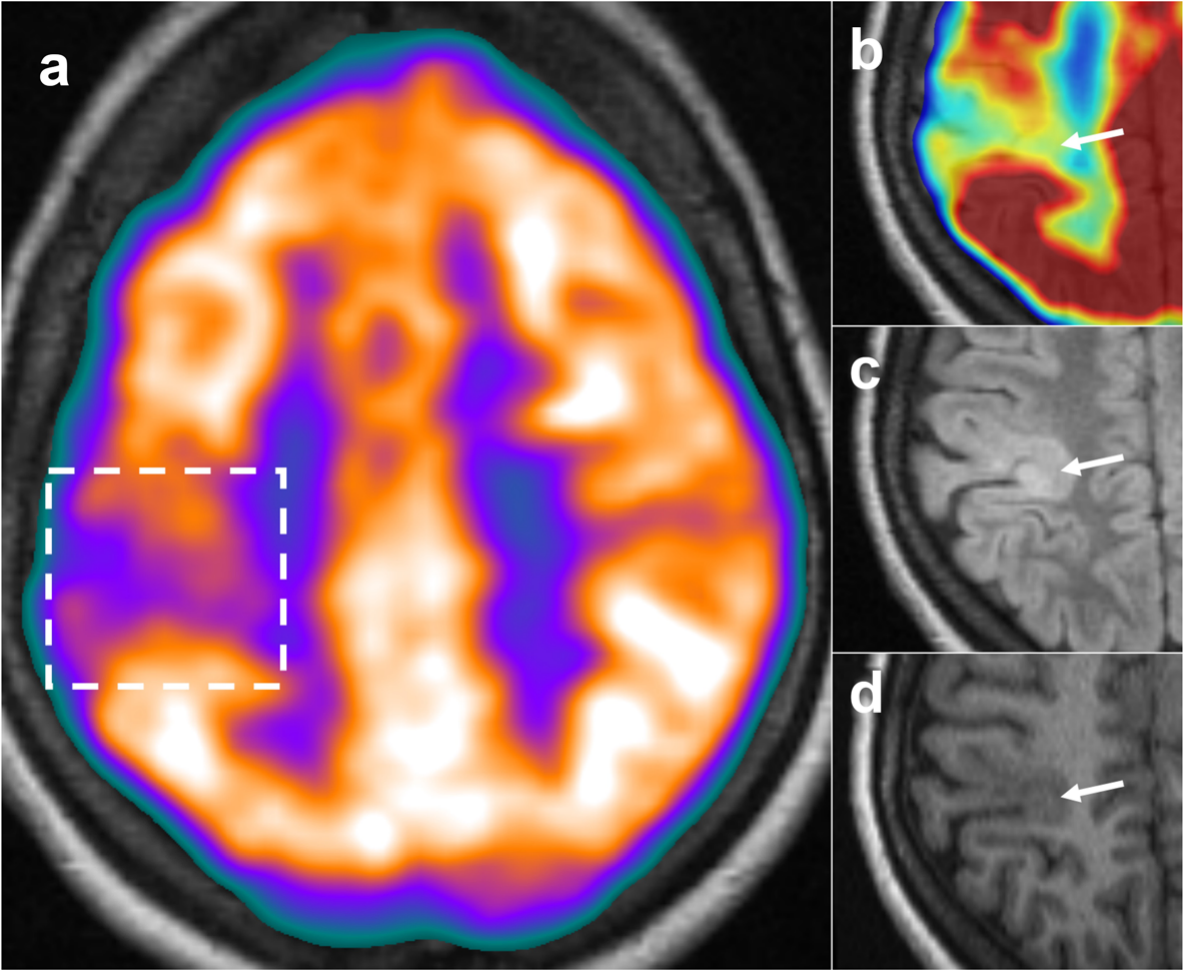
Appearance of BOSD on PET and MRI. (**a**) Coregistered PET and MRI data is shown for one patient who exhibited BOSD MRI hallmarks, as well as focal hypometabolism on ^18^F-FDG-PET. The area of focal hypometabolism is shown in the dashed box. The area of PET hypometabolism is shown in an inset area in (**b**) using a jet colour scheme. The FLAIR image (**c**) shows T2 hyperintensity of the BOSD, while this appears as a hypointensity on T1 (**d**)

**Fig. 2:**
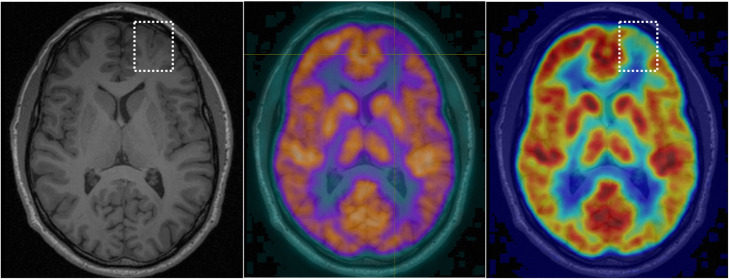
MRI and PET images for an individual with focal PET hypometabolism. A subtle BOSD was detected on MRI in this patient. The structural abnormality is shown in the left image on T1 in the dotted box. ^18^F-FDG PET also showed an area of focal hypometabolism (middle: co-registered PET overlaid on T1 with crosshairs on structural BOSD; right: PET image in jet colour scheme with dotted box on structural abnormality)

**Fig. 3:**
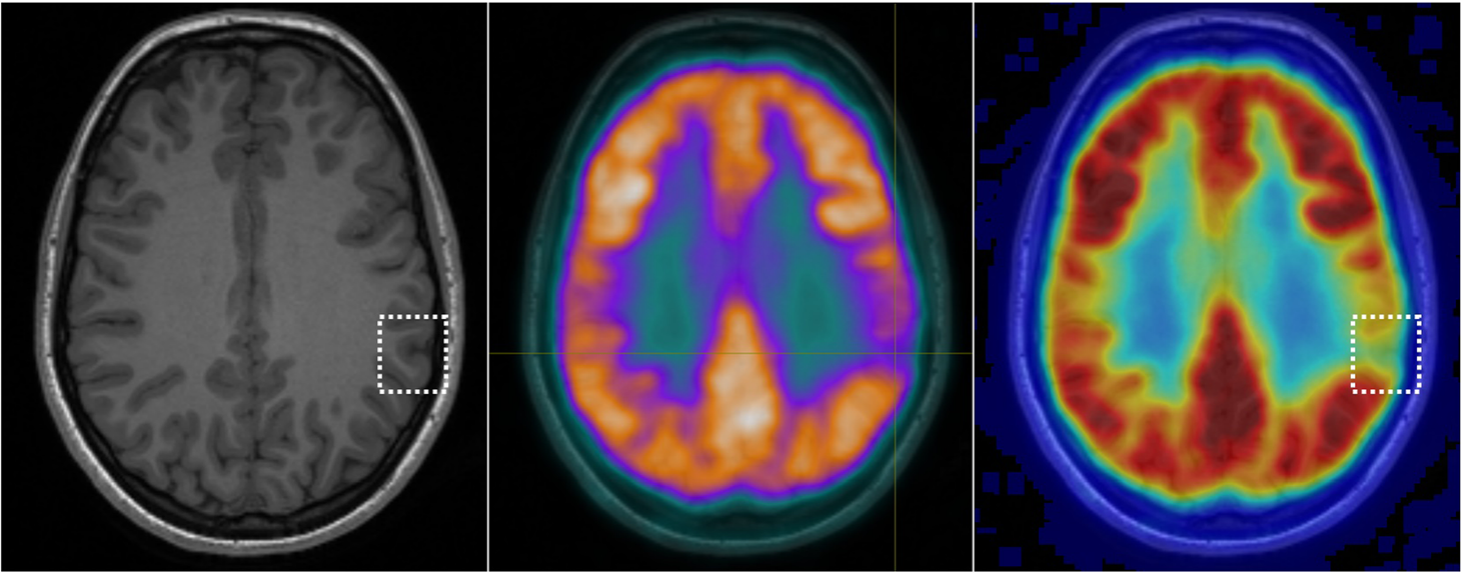
MRI and PET images for an individual with focal PET hypometabolism enabling detection of a subtle BOSD. MRI was initially negative in this patient, while a focal hypometabolism was detected on PET. Subsequent coregistration of MRI and PET enabled detection of a small BOSD in this patient. This patient was seizure free following focal resection of the subtle BOSD

**Fig. 4:**
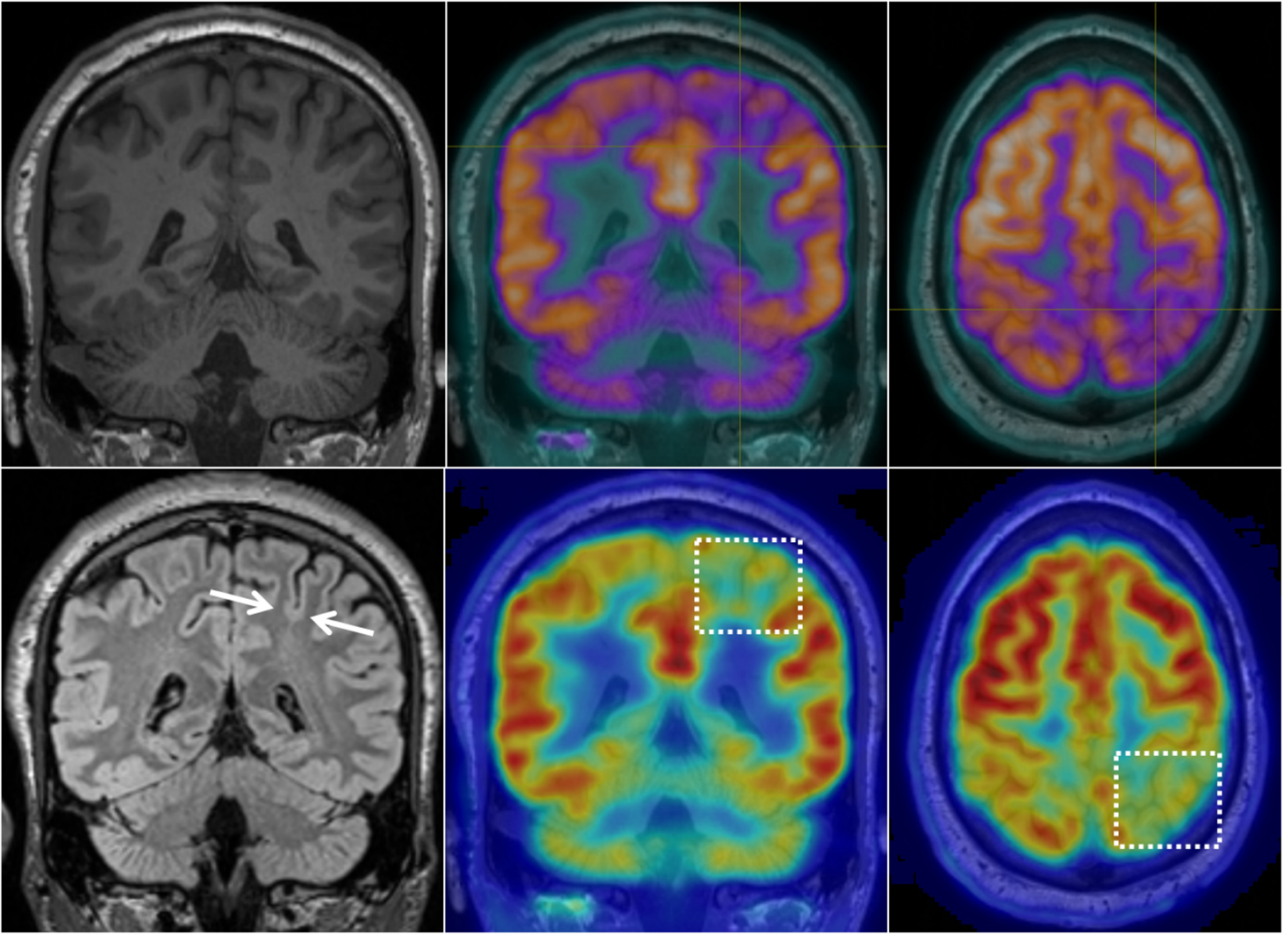
MRI and PET images for an individual with regional PET hypometabolism. Regional hypometabolism was evident in this patient, with gradual decline in activity evident within the boxed area when compared to adjacent cortex. MRI was initially negative, and a subtle BOSD was detected only upon the second review of MRI with co-registered PET in this patient (arrows shown on FLAIR in bottom left image). This patient was seizure free following focal resection of the BOSD

PET was positive in 19/20 cases (95%), with only one patient classified as PET negative. A focal abnormality was detected in 12/20 (60%) of patients, while regional hypometabolism was evident in 7/20 (35%) patients (see Figs. 1, 2, 3, and 4 for examples). In all cases where a PET abnormality was identified (19 patients), the final seizure focus location was concordant with MRI findings. In the one PET-negative patient, subsequent review of the PET image with co-registered MRI showed a reduction in cortical metabolic activity corresponding to the MRI abnormality.

In the four MRI-negative patients, a second review of MRI with co-registration to PET enabled the identification of subtle abnormalities in all patients that were previously missed upon initial examination. Table 2 shows clinical data in relation to MRI findings.

**Table 2:** PET data in relation to MRI and surgical results

	Initial MRI positive (*n* = 16)	Initial MRI negative (*n* = 4)
PET visual analysis		
Focal hypometabolism	10	2
Regional hypometabolism	5	2
No PET abnormality	1	0
BOSD location		
Frontal	9	2
Parietal	4	2
Frontotemporal	1	0
Temporoparietal	1	0
Insula	1	0
Histopathology		
FCD type IIA	1	1
FCD type IIB	15	2
Negative pathology	0	1
Post-surgical outcome		
Seizure free	13	4
Seizure reduction	1	0
Recurrent seizures	2	0

### Histopathology

Pathology of the surgical specimen confirmed FCD type IIB in 17/20 patients (85%) and FCD type IIA in two patients (10%). Negative pathology was reported in one patient, despite characteristic clinical and imaging features of BOSD. In this patient, the BOSD was a highly focal lesion in eloquent left parietal cortex, and the negative pathology result was likely a reflection of the limited pathological specimen due to the very small size of the resected area.

Concerning the ^18^F-FDG PET findings, of the 12 patients exhibiting focal PET hypometabolism, all but one exhibited FCD type IIB pathology (19/20; 95%), while the one remaining patient was the individual with negative pathology. Of the seven patients exhibiting regional hypometabolism, two exhibited FCD type IIA pathology (29%), while the remaining five exhibited type IIB pathology (71%).

### Surgical outcome

In 17 of 20 patients, seizure freedom was achieved with a mean follow-up period of 670 days (in one case after a third surgical procedure). Of the remaining three patients, seizure frequency was reduced in one, while two patients had recurrent seizures, with one experiencing seizures in the immediate post-surgical period.

When taking into account the ^18^F-FDG PET findings, of the 12 patients exhibiting a focal abnormality, 11/12 (92%) experienced complete seizure freedom in the follow-up period. Of the seven patients exhibiting a regional hypometabolic abnormality on FDG-PET, 5/7 (71%) experienced complete seizure freedom in the follow-up period, while 2 patients experienced recurrent seizures, which were infrequent in one case.

## Discussion

In this study, we explored the contribution of ^18^F-FDG PET in the detection of BOSD lesions, alongside MRI. In our cohort of 20 BOSD patients, we found that ^18^F-FDG PET exhibited focal or regional abnormality consistent with the dysplastic lesion in 95% of cases, contributing remarkably to the diagnosis of BOSD.

Initial review of MR images missed the dysplastic lesion in 20% of cases, while co-registration with PET enabled detection of the BOSD in all cases. The relatively high sensitivity of MRI to detect BOSD lesions in our cohort may relate to improved detection with 3 T MRI and better acquisition methods when compared to previous studies in which negative MRIs have been commonly reported (Salamon et al. 2008; Chassoux et al. 2010). Nonetheless, ^18^F-FDG PET was more sensitive than MRI in detecting hypometabolic abnormalities consistent with the dysplastic lesion, at least upon initial review, detecting an abnormality in 95% of cases. Indeed, when comparing the prognostic value of MRI and ^18^F-FDG PET, PET has been shown to be marginally better than MRI in the detection of FCD type IIB lesions, though not for FCD type IIA (Kim et al. 2009).

In this study, the added value of PET was particularly evident in those cases where MRI was initially negative. Following coregistration of PET/MRI, a subtle BOSD was detected in all cases where MRI was initially negative (see Fig. 4). Indeed, others similarly report the high localising value of ^18^F-FDG PET following PET/MRI coregistration in FCD, particularly in cases where MRI alone was negative or doubtful (Salamon et al. 2008; Chassoux et al. 2010; Desarnaud et al. 2018). The inclusion of PET in presurgical workup is indeed valuable in localising these small BOSD lesions, in line with findings from FCD cohorts.

Despite the localised nature of small BOSD lesions, the PET metabolic abnormality was not necessarily focal in nature across all patients. While the majority of patients exhibited focal hypometabolism, 35% of patients exhibited regional hypometabolism that extended beyond the focal structural lesion. All but one of the patients with focal PET hypometabolism exhibited FCD type IIB pathology, with the one remaining patient being pathology negative, likely due to limited tissue sample in this previously described case (Jackson et al. 2017). In contrast, those with regional PET hypometabolism included cases exhibiting FCD type IIA pathology, in which the abnormality was more complex and extended beyond the visible BOSD.

Localisation of these small epileptogenic lesions is highly important, as removal of the focal lesion results in an excellent post-surgical outcome. In our cohort, a successful surgical outcome was high, with 87% of cases being seizure-free in the follow-up period. This was in line with a previous study in an overlapping BOSD cohort (Harvey et al. 2015), as well as in other FCD cohorts with type II pathology (Chassoux et al. 2010). In previous FCD studies, the primary predictor of unfavourable surgical outcome is reported to be incomplete removal of the dysplastic lesion (Kim et al. 2009; Krsek et al. 2009; Rowland et al. 2012). Accurately identifying and removing the dysplastic lesion is thus of crucial importance. In our cohort, the BOSD was identified on MRI in all three patients who experienced recurrent seizures following surgery; however, it was interesting to note that of the three patients, two exhibited regional, rather than focal, hypometabolism on ^18^F-FDG PET. It could be that the epileptogenic zone in these two participants extended beyond the structural abnormality that was removed, and that surgical removal was incomplete. Indeed, widespread connectivity disruptions have been reported beyond the focal lesion, in both drug-resistant localization-related epilepsy and pathologically confirmed type II FCD cohorts (Besson et al. 2017; Hong et al. 2019). However, it should be noted that PET has previously shown lower spatial resolution than functional connectivity measures, and the area needed to be resected is likely smaller than the area of PET hypometabolism (Jackson et al. 2017).

There are limitations to this study that should be highlighted. This was a small cohort study, and our estimates of the sensitivity of PET in detecting BOSDs are limited by the small sample size. Moreover, given the retrospective nature of this study, our inclusion criteria limited the study cohort to those in whom a BOSD was identified and who then proceeded to surgery, and we cannot assess the potential false-negative cases who were not referred to surgery. Another limitation to our work relates to the PET analysis, which in this study was limited to visual assessments, given its integration into routine pre-surgical workup. There are various quantitative techniques, including post-processing methods and machine-learning approaches, that have recently exhibited improved detection of FCD lesions (Tan et al. 2018; Liu et al. 2019) and improved detection of BOSD (Besson et al. 2008). Future work could benefit from investigating quantitative methods, particularly those combining MRI and PET, in the detection and associated surgical outcomes in BOSD. Finally, while we focus here on the contribution of ^18^F-FDG PET in the clinical workup for BOSD, it may be interesting in future to assess the relationship and additional value of electrophysiological data, as has been investigated in other FCD cohorts (Lagarde et al. 2016; Chassoux et al. 2012).

In conclusion, the findings of this work suggest that ^18^F-FDG PET enables excellent sensitivity in the detection of BOSDs and contributes additional localising value, particularly when there is difficulty in detecting these lesions with MRI. The detection of focal PET hypometabolism in particular is associated with excellent post-surgical outcome, and our findings support the routine use of PET in presurgical workup in patients with a suspected BOSD.

## Data Availability

The clinical and imaging data are not publicly available due to ethical restrictions with patient data.

## Abbreviations

BOSD: Bottom-of-sulcus dysplasia
CEP: Comprehensive Epilepsy Program
EEG: Electroencephalography
FCD: Focal cortical dysplasia
18F-FDG: 18F-fluorodeoxyglucose
FLAIR: Fluid-attenuated inversion recovery
